# Supplementary material for: Old lake versus young taxa: a comparative phylogeographic perspective on the evolution of Caspian Sea gastropods (Neritidae: Theodoxus)
Source: R Soc Open Sci. 2019 Oct 16;6(10):190965. doi: 10.1098/rsos.190965 (PMC6837181; doi:10.1098/rsos.190965)
Supplement: Appendix B [file rsos190965supp2.docx]

*Royal Society Open Science*

**Appendix B**

Old lake vs. young taxa: a comparative phylogeographic perspective on the evolution of Caspian Sea gastropods (Neritidae: *Theodoxus*)

Arthur F. Sands, Thomas A. Neubauer, Saeid Nasibi, Majid Fasihi Harandi, Vitaliy V. Anistratenko, Thomas Wilke, Christian Albrecht

**Appendix B:**

Supporting information: Results from db-RDA and associated pre-analyses

(calculated in R vs. 3.5.2 with package ‘vegan’ vs. 2.5-4)

**Supporting information: Results from db-RDA and associated pre-analyses**

(calculated in R vs. 3.5.2 with package ‘vegan’ vs. 2.5-4)

**1. Pontocaspian group**

***1.1 Correlation between latitude, longitude and geographic distance (GGD)***

**Table 1.1.1.** Correlation between latitude, longitude and geographic distance (GGD) for mtDNA markers (COI and 16S).

|  | Latitude | Longitude | GGD |
| --- | --- | --- | --- |
| Latitude | 1 | -0.6298383 | -0.8676993 |
| Longitude | -0.6298383 | 1 | 0.9323556 |
| GGD | -0.8676993 | 0.9323556 | 1 |

**Table 1.1.2.** Correlation between latitude, longitude and geographic distance (GGD) for phased nDNA (ATPα).

|  | Latitude | Longitude | GGD |
| --- | --- | --- | --- |
| Latitude | 1 | -0.6298375 | -0.8676915 |
| Longitude | -0.6298375 | 1 | 0.9323613 |
| GGD | -0.8676915 | 0.9323613 | 1 |

***1.2 Results of db-RDA for COI (genetic distance (GD) vs. GGD)***

**Table 1.2.1.** Results db-RDA – COI (GD vs. GGD).

|  | Inertia | Proportion | Rank |
| --- | --- | --- | --- |
| Total | 162.8 | 1.000 |  |
| Constrained | 1.115 | 0.006846 | 1 |
| Unconstrained | 161.7 | 0.9932 | 173 |

**Table 1.2.2.** Eigenvalues for constrained unconstrained axes.

|  | Axes | Eigenvalues |
| --- | --- | --- |
| Constrained axes | CAP1 | 1.1149 |
| Unconstrained axes  (first 8 of 173 axes) | MDS1 | 17.669 |
|  | MDS2 | 1.701 |
|  | MDS3 | 1.513 |
|  | MDS4 | 1.010 |
|  | MDS5 | 0.907 |
|  | MDS6 | 0.876 |
|  | MDS7 | 0.868 |
|  | MDS8 | 0.852 |

Constant added to distances: 0.8307744

**Table 1.2.3.** Results of the permutation test for capscale under reduced model, where permutations were free and the number of permutations = 9999.

|  | Df | Variance | F | Pr(>F) |
| --- | --- | --- | --- | --- |
| Model | 1 | 1.115 | 1.1926 | 0.0882 |
| Residual | 173 | 161.732 |  |  |

**Table 1.2.4.** R² and adjusted R² values for the for COI dataset (GD vs. GGD).

|  | Coefficient of determination |
| --- | --- |
| R² | 0.006846358 |
| R²_adj_ | 0.001105585 |

***1.3 Results of db-RDA for 16S (GD vs. GGD)***

**Table 1.3.1.** Results db-RDA – 16S (GD vs. GGD).

|  | Inertia | Proportion | Rank |
| --- | --- | --- | --- |
| Total | 14.61307 | 1.000 |  |
| Constrained | 0.20130 | 0.01378 | 1 |
| Unconstrained | 14.41176 | 0.98622 | 173 |

**Table 1.3.2.** Eigenvalues for constrained unconstrained axes.

|  | Axes | Eigenvalues |
| --- | --- | --- |
| Constrained axes | CAP1 | 0.2013 |
| Unconstrained axes  (first 8 of 173 axes) | MDS1 | 0.8416 |
|  | MDS2 | 0.2239 |
|  | MDS3 | 0.1089 |
|  | MDS4 | 0.0968 |
|  | MDS5 | 0.0896 |
|  | MDS6 | 0.0896 |
|  | MDS7 | 0.0894 |
|  | MDS8 | 0.0781 |

Constant added to distances: 0.0781088

**Table 1.3.3.** Results of the permutation test for capscale under reduced model, where permutations were free and the number of permutations = 9999.

|  | Df | Variance | F | Pr(>F) |
| --- | --- | --- | --- | --- |
| Model | 1 | 0.2013 | 2.4164 | 0.0001 |
| Residual | 173 | 14.4118 |  |  |

**Table 1.3.4.** R² and adjusted R² values for the for 16S dataset (GD vs. GGD).

|  | Coefficient of determination |
| --- | --- |
| R² | 0.0137754 |
| R²_adj_ | 0.008074681 |

***1.4 Results of db-RDA for ATPα (GD vs. GGD)***

**Table 1.4.1.** Results db-RDA – ATPα (GD vs. GGD).

|  | Inertia | Proportion | Rank |
| --- | --- | --- | --- |
| Total | 57.591485 | 1.000 |  |
| Constrained | 0.218368 | 0.003792 | 1 |
| Unconstrained | 57.373117 | 0.996208 | 348 |

**Table 1.4.2.** Eigenvalues for constrained unconstrained axes.

|  | Axes | Eigenvalues |
| --- | --- | --- |
| Constrained axes | CAP1 | 0.21837 |
| Unconstrained axes  (first 8 of 173 axes) | MDS1 | 0.9065 |
|  | MDS2 | 0.4574 |
|  | MDS3 | 0.3264 |
|  | MDS4 | 0.2343 |
|  | MDS5 | 0.1975 |
|  | MDS6 | 0.1781 |
|  | MDS7 | 0.1685 |
|  | MDS8 | 0.1658 |

Constant added to distances: 0.1614122

**Table 1.4.3.** Results of the permutation test for capscale under reduced model, where permutations were free and the number of permutations = 9999.

|  | Df | Variance | F | Pr(>F) |
| --- | --- | --- | --- | --- |
| Model | 1 | 0.218 | 1.3245 | 0.0001 |
| Residual | 348 | 57.373 |  |  |

**Table 1.4.4.** R² and adjusted R² values for the for ATPα dataset (GD vs. GGD).

|  | Coefficient of determination |
| --- | --- |
| R² | 0.00379167 |
| R²_adj_ | 0.0009290029 |

**2. Southern Iranian group**

***2.1 Correlation between latitude, longitude and geographic distance (GGD)***

**Table 2.1.1.** Correlation between latitude, longitude and geographic distance (GGD) for mtDNA markers (COI and 16S).

|  | Latitude | Longitude | GGD |
| --- | --- | --- | --- |
| Latitude | 1 | -0.7901439 | 0.9137417 |
| Longitude | -0.7901439 | 1 | -0.9710101 |
| GGD | 0.9137417 | -0.9710101 | 1 |

**Table 2.1.2.** Correlation between latitude, longitude and geographic distance (GGD) for phased nDNA (ATPα).

|  | Latitude | Longitude | GGD |
| --- | --- | --- | --- |
| Latitude | 1 | -0.7901439 | 0.9137414 |
| Longitude | -0.7901439 | 1 | -0.9710102 |
| GGD | 0.9137414 | -0.9710102 | 1 |

***2.2 Results of db-RDA for COI (GD vs. GGD)***

**Table 2.2.1.** Results db-RDA – COI (GD vs. GGD).

|  | Inertia | Proportion | Rank |
| --- | --- | --- | --- |
| Total | 98.9103 | 1.000 |  |
| Constrained | 19.6688 | 0.1989 | 1 |
| Unconstrained | 79.2415 | 0.8011 | 43 |

**Table 2.2.2.** Eigenvalues for constrained unconstrained axes.

|  | Axes | Eigenvalues |
| --- | --- | --- |
| Constrained axes | CAP1 | 19.669 |
| Unconstrained axes  (first 8 of 173 axes) | MDS1 | 63.47 |
|  | MDS2 | 14.70 |
|  | MDS3 | 0.32 |
|  | MDS4 | 0.02 |
|  | MDS5 | 0.02 |
|  | MDS6 | 0.02 |
|  | MDS7 | 0.02 |
|  | MDS8 | 0.02 |

Constant added to distances: 0.0192652

**Table 2.2.3.** Results of the permutation test for capscale under reduced model, where permutations were free and the number of permutations = 9999.

|  | Df | Variance | F | Pr(>F) |
| --- | --- | --- | --- | --- |
| Model | 1 | 19.669 | 10.673 | 0.0004 |
| Residual | 43 | 79.241 |  |  |

**Table 2.2.4.** R² and adjusted R² values for the for COI dataset (GD vs. GGD).

|  | Coefficient of determination |
| --- | --- |
| R² | 0.1988552 |
| R²_adj_ | 0.1802239 |

***2.3 Results of db-RDA for 16S (GD vs. GGD)***

**Table 2.3.1.** Results db-RDA – 16S (GD vs. GGD).

|  | Inertia | Proportion | Rank |
| --- | --- | --- | --- |
| Total | 10.234 | 1.000 |  |
| Constrained | 4.974 | 0.486 | 1 |
| Unconstrained | 5.260 | 0.514 | 3 |

**Table 2.3.2.** Eigenvalues for constrained unconstrained axes.

|  | Axes | Eigenvalues |
| --- | --- | --- |
| Constrained axes | CAP1 | 4.974 |
| Unconstrained axes | MDS1 | 3.328 |
|  | MDS2 | 1.866 |
|  | MDS3 | 0.066 |

Constant added to distances: 0.000000000000001406526

**Table 2.3.3.** Results of the permutation test for capscale under reduced model, where permutations were free and the number of permutations = 9999.

|  | Df | Variance | F | Pr(>F) |
| --- | --- | --- | --- | --- |
| Model | 1 | 4.9742 | 40.663 | 0.0001 |
| Residual | 43 | 5.2601 |  |  |

**Table 2.3.4.** R² and adjusted R² values for the for 16S dataset (GD vs. GGD).

|  | Coefficient of determination |
| --- | --- |
| R² | 0.4860327 |
| R²_adj_ | 0.47408 |

***2.4 Results of db-RDA for ATPα (GD vs. GGD)***

**Table 2.4.1.** Results db-RDA – ATPα (GD vs. GGD).

|  | Inertia | Proportion | Rank |
| --- | --- | --- | --- |
| Total | 20.93606 | 1.000 |  |
| Constrained | 1.43963 | 0.06876 | 1 |
| Unconstrained | 19.49644 | 0.93124 | 88 |

**Table 2.4.2.** Eigenvalues for constrained unconstrained axes.

|  | Axes | Eigenvalues |
| --- | --- | --- |
| Constrained axes | CAP1 | 1.4396 |
| Unconstrained axes  (first 8 of 173 axes) | MDS1 | 4.460 |
|  | MDS2 | 0.714 |
|  | MDS3 | 0.360 |
|  | MDS4 | 0.343 |
|  | MDS5 | 0.246 |
|  | MDS6 | 0.185 |
|  | MDS7 | 0.163 |
|  | MDS8 | 0.163 |

Constant added to distances: 0.1627528

**Table 2.4.3.** Results of the permutation test for capscale under reduced model, where permutations were free and the number of permutations = 9999.

|  | Df | Variance | F | Pr(>F) |
| --- | --- | --- | --- | --- |
| Model | 1 | 1.4396 | 6.498 | 0.0001 |
| Residual | 88 | 19.4964 |  |  |

**Table 2.4.4.** R² and adjusted R² values for the for ATPα dataset (GD vs. GGD).

|  | Coefficient of determination |
| --- | --- |
| R² | 0.06876303 |
| R²_adj_ | 0.05818079 |
